# Morphometric divergence in populations of *Anastrepha obliqua* (Diptera, Tephritidae) from Colombia and some Neotropical locations

**DOI:** 10.3897/zookeys.540.6013

**Published:** 2015-11-26

**Authors:** Maria R. Castañeda, Denise Selivon, Vicente Hernández-Ortiz, Alberto Soto, Nelson A. Canal

**Affiliations:** 1 Universidad del Tolima, Barrio Altos de Santa Helena, Ibagué, Tolima, Colombia, CP 73000629; 2 Doctorado en Ciencias Agrarias, Universidad de Caldas, Manizales, Carrera 35 62-160, sede Sancancio, Manizales, Caldas, Colombia; 3 Departamento de Biologia, Instituto de Biociências, Universidade de São Paulo, 05508-900 São Paulo, São Paulo, Brazil; 4 Instituto de Ecología A.C., Red de Interacciones Multitróficas. Carretera antigua a Coatepec # 351, El Haya. Xalapa, Veracruz 91070, México

**Keywords:** West Indian fruit fly, taxonomy, linear morphometry

## Abstract

The West Indian fruit fly, *Anastrepha obliqua*, is one of seven species of quarantine importance of its genus and is one of the most economically important fruit fly pests in Colombia. The taxonomic status of this species is a key issue for further implementation of any pest management program. Several molecular studies have shown enough variability within *Anastrepha obliqua* to suggest its taxonomic status could be revised; however, there are no morphological studies supporting this hypothesis. The aim of this work was to describe the morphological variability of Colombian populations of *Anastrepha obliqua*, comparing this variability with that of other samples from the Neotropics. Measurements were performed on individuals from 11 populations collected from different geographic Colombian localities and were compared with populations from Mexico (2), Dominica Island (1), Peru (1) and Brazil (2). Linear morphometric analyses were performed using 23 female morphological traits, including seven variables of the aculeus, three of the thorax, and six of the wing; seven ratios among them were also considered. Discriminant function analyses showed significant morphological differentiation among the Colombian populations, separating them into two groups. Furthermore, in the comparisons between Colombian samples with those from other countries, three clusters were observed. The possibility of finding more than one species within the nominal *Anastrepha obliqua* population is discussed.

## Introduction

*Anastrepha* Schiner is the most diverse and economically important genus of fruit flies in the Neotropics, with more than 250 described species (Norrbom et al. 2012). *Anastrepha obliqua* (Macquart), known as the West Indian fruit fly, has quarantine status and is one of the most harmful species within the genus (Norrbom et al. 2012, Ruíz-Arce et al. 2012). It is distributed from northern Mexico to southeast Brazil including several Caribbean islands (Ruíz-Arce et al. 2012). Norrbom (2004) lists 104 species within 27 host plant families for *Anastrepha obliqua*; however, the species most affected by this pest are mango (*Mangifera indica*) and *Spondias* in Mexico (Orozco-Dávila et al. 2014), Colombia (Nuñez 1981, Mangan et al. 2011), the southern Caribbean (Mangan et al. 2011) and Brazil (Zucchi 2000).

The West Indian fruit fly belongs to the *fraterculus* group, which involves 34 species (Norrbom et al. 2012) from which one complex of cryptic species has been recognized (Hernández-Ortiz et al. 2012). The taxonomic status of *Anastrepha obliqua* has been questioned, and the existence of a group of cryptic species within the nominal species is presumed (Ruíz-Arce et al. 2012). Hernández-Ortiz et al. (2004, 2012, 2015) studied Latin American populations of *Anastrepha fraterculus* (Wiedemann) using linear morphometry of the aculeus and the wings and found eight different morphotypes, showing that the use of morphological characters and linear morphometry is a useful tool for the study of cryptic species in the genus *Anastrepha*, especially in the *fraterculus* group.

Smith-Caldas et al. (2001) studied the COI mitochondrial gene sequences of 15 species of *Anastrepha*, 12 of which were from the *fraterculus* group. The sample included eight populations of *Anastrepha obliqua*: five from Brazil, two from Mexico and one from Colombia. The results showed two well-separated groups, the first of which included the Mexican population, the Colombian population and one Brazilian population while the remaining Brazilian populations formed a second group. Therefore, the authors indicated the need to study this species more extensively. Ruiz-Arce et al. (2012) studied 54 Latin American populations of *Anastrepha obliqua* with COI and ND6 genes and concluded that there are six genetic types that require taxonomic revision (Mesoamerica, Central America, Caribbean, Western Mexico, South American Andes and Eastern Brazil).

Other studies have also suggested variability within the species. Karyotype descriptions of Brazilian and Mexican samples reflect the existence of a constriction at the apical end of the X chromosome (Solferini and Morgante 1987, Selivon et al. 2005, Ibañez-Palacios et al. 2010), while Bush (1962) studied individuals that were clearly devoid of that chromosome constriction. Additionally, morphological descriptions of eggs (Emmart 1933, Norrbom 1985, Murillo and Jiron 1994, Selivon and Perondini 2000, Figuereido et al. 2011) and larvae (White and Elson-Harris 1992, Frías et al. 2006) also reported morphological variability in the populations studied.

In Colombia, mango is the second most important fruit product based on its planting area and it is cultured at low altitudes throughout the country. *Anastrepha obliqua* is one of the biggest limiting factors of its production (MADR 2006, Sosa et al. 2011), and therefore, the development of sustainable management systems has become a priority. This species of fruit fly is widely distributed at altitudes less than 1,500 m, following the distribution of mango and the species of *Spondias*, which are its main hosts (Castañeda et al. 2010). Because the Andes of Colombia are divided into three high altitude mountain ranges, extending east to Venezuela with two deep valleys separating them, the populations of *Anastrepha obliqua* located in lowlands could be isolated in the different areas of the country.

The Sterile Insect Technique (SIT), for the control of the West Indian fruit fly” has been implemented in Mexico, and this fly is one of the priority species for the development of this technique in other regions (Cáceres et al. 2014, Orozco-Dávila et al. 2014). Studies to identify more efficient specific attractants are also being developed (Cruz-López et al. 2006). However, the precise taxonomic knowledge of the pest is a fundamental requirement for these techniques, as well as other quarantine measures, risk analysis and free or low prevalence areas (Hernández-Ortiz et al. 2004, Norrbom et al. 2012).

The study of the interpopulation variability of *Anastrepha obliqua* in Colombia or other areas of the Neotropics is particularly relevant to determine if this nominal species is composed by a cryptic species complex considering the implications this has for fruit international trade. These studies are also important for the implementation of pest management systems in different regions, such as the SIT. The aim of this study was to describe the morphologic variability of *Anastrepha obliqua* through the use of multivariate morphometric analyzes among Colombian populations distributed throughout the country and through comparisons with populations coming from its distribution range such as Mexico, Peru, Brazil and Dominica Island. The hypotheses of this study is that if there is genetic variation in *Anastrepha obliqua* suggesting the existence of more than a simple biological entity (Ruiz-Arce et al. 2012), this variability must be observed in the morphology of the species.

## Methodology

### Insect collection

**Figure 1.**
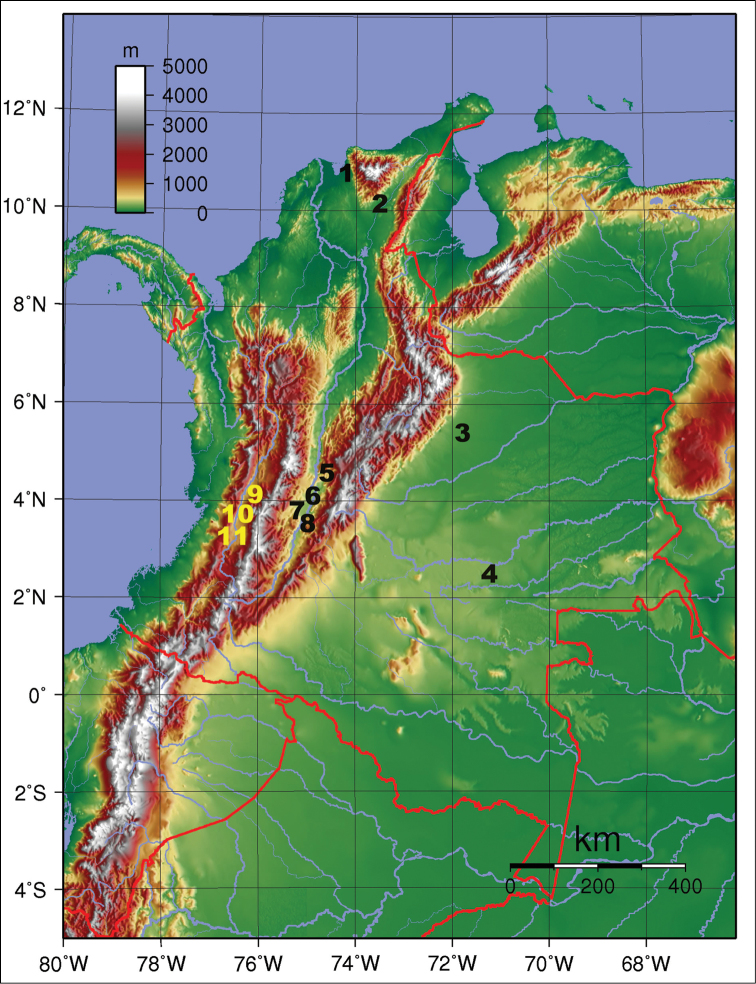
Geographic distribution of populations of *Anastrepha obliqua* collected in Colombia for morphometric analysis: **1** Cienaga **2** Huacachi **3** Villanueva **4** Puerto Colombia **5** Anolaima **6** Coello **7** Espinal, 8 Guamo **9** La Tebaida **10** Zarzal, and **11** La Unión. (Image from https://elpaisdelcafe.files.wordpress.com/2012/02/200px-colombia_topography.png)

The individuals studied were collected from 11 localities of Colombia along the inter-Andean valleys of the Magdalena River (4) and Cauca River (3) and in the Eastern Plains (2) and the Caribbean Plains (2) (Figure 1). These populations were compared with two samples from Mexico (East and West), two from southeastern Brazil (Sao Paulo), one from Peru and one from Dominica Island (Table 1). Specimens were collected from infested fruits or McPhail traps placed in the field or obtained from laboratory colonies. Samples of mango fruit (*Mangifera indica*) and several species of *Spondias* were collected. The fruits were collected in plastic trays with damp vermiculite and transferred to the laboratory, where they remained for the separation of larvae/pupae and subsequent acquisition of adults. The imagos were kept in cages and fed for at least five days for the fixation of their morphological characteristics. In order to obtain native populations of insects, fruit sampling was conducted in non-commercial areas. Specimens from McPhail traps were collected from only one trap in the same date. Samples from Espinal and Guamo (Colombia) were obtained from a laboratory colony maintained, in artificial diet, since 2005 with periodical introductions of wild flies. In these cases, specimens were obtained three generations after field specimens were introduced in the colony. Samples from Brazil were obtained from specimens stored in alcohol from reared insects at two different times and came from colonies reared in fruits for a few generations.

**Table 1.** Collection data for *Anastrepha obliqua* populations.

Country	Population	State	City	Host	Data	Latitude	Longitude	Altitude	n
Colombia	Anolaima	Cundinamarca	Anolaima	*Mangifera indica*	6/8/2010	4°43'23"N	74°25'4"W	972	20
	Coello	Tolima	Coello	*Spondias mombin*	8/28/2010	4°16'55"N	74°54'16"W	309	20
	Guamo		Guamo	*Spondias purpurea*	Lab rearing	4°04'35"N	74°59'35"W	345	20
	Espinal		Espinal	*Mangifera indica*	Lab rearing	4°11'59"N	74°58'0.7"W	380	20
	La Union	Valle del Cauca	La Unión	*Mangifera indica*	11/12/2013	4°33'53"N	76°05'22"W	954	14
	Zarzal		Zarzal	*Mangifera indica*	11/11/2013	4°25'33"N	76°03'43"W	954	19
	La Tebaida	Quindio	La tebaida	*Spondia* sp.	7/10/2010	4°29'42"N	75°41'36"W	1409	20
	Villanueva	Casanare	Villanueva	*Spondia* sp.	3/22/2013	4°22'13"N	72°46'17"W	160	20
	PuertoC	Guaviare	Purto Colombia	McPhail Traps	10/09/09	2°36'13"N	72°39'W	189	20
	Huacachi	Cesar	Huacachi	*Mangifera indica*	8/6/2013	10°30'25"N	73°0.9'47"W	136	20
	Cienaga	Magdalena	Cienaga	McPhail Traps	8/1/2005	11°58'92"N	74°12'18"W	N.R	18
México	Mex-Pacific	Guerrero	Los Ayutlas	McPhail Traps	06/28/2008	16°59'17"N	99°04'57"W	340	20
	Mex-Gulf	Veracruz	Los Tuxtlas	*Spondias* sp	10/19/2012	18°26'36"N	95°02'46"W	397	20
Brasil	Brazil-1	São Paulo	USP	*Spondias mombin*	Lab rearing	23°33'55"S	46°44'04"W	780	9
	Brazil-2	São Paulo	USP	*Spondias mombin*	Lab rearing	23°33'55"S	46°44'04"W	780	10
Peru	Peru-Pacific			McPhail Traps	ND	ND	ND	ND	20
Isla Dominica	Caribe			McPhail Traps	ND	15°31'24"N	61°21'56"W	ND	17

### Morphological variables

Morphometric studies were conducted on the aculeus, right wing and mesonotum of randomly selected five-day-old females following methods for the study of the *Anastrepha fraterculus* complex described by Hernández-Ortiz et al. (2004, 2012). The ovipositor was cleaned with 10% sodium hydroxide for 24 hours and the wing and the aculeus were mounted on permanent slides with Canad balsam. The abdomen and thorax were preserved in 70% alcohol. The aculeus was photographed with a Canon Powershot G10 digital camera, adapted to a Carl Zeiss Primo Star microscope.

The wing and the mesonotum were photographed with a Moticam10X camera adapted to a stereomicroscope. The images were measured with the software Motic Image Plus 2.0 (Motic 2013); all measurements were performed by only one observer (MRC).

**Figure 2.**
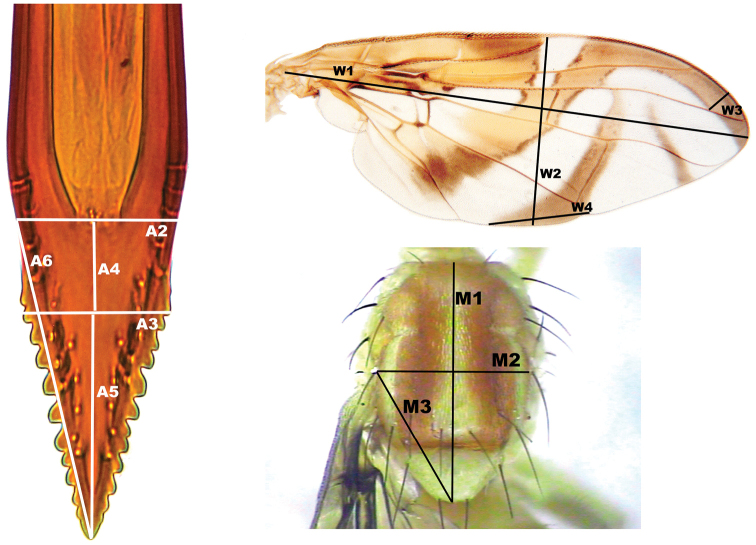
Diagram of the structures and variables used in the morphometric analysis of *Anastrepha obliqua* females in the aculeus (ventral view), mesonotum (dorsal view) and wing. The variables are described in the text.

For the study, 23 variables were used (Figure 2): *Aculeus*: A1, total length of the aculeus; A2, width at the end of the sclerotized margin on the ventral side; A3, width at the beginning of the serrated section, measured between the apices of the second pair of teeth; A4, length of the basal end of the aculeus (from the margin of the sclerotized area on the ventral side at the beginning of the serrated section); A5, length of the apex of the aculeus (length of the serrated section); A6, length of the lateral right side from the base of the sclerotized area; A7, average number of teeth per side; A8, length of the apex (A4+A5); A9, proportion of the non-serrated and serrated areas of the apex (A4/A5); A10, proportion of the length of the tip of the aculeus and total aculeus length (A8/A1); and A11, proportion of the non-serrated area of the apex and total apex length (A4/A8). *Wing*: W1, length of the wing from the basal extreme of the costal margin to the apex; W2, wing width at the apex of the R1 vein; W3, width of the S band from the union of the S band and the R4+5 vein perpendicular to the costa; W4, width of the base of the proximal branch of the V band; W5, S- and V-band connection between R_2+3_ and R_4+5_ (1 = present, 2 = absent); W6, V-band anterior connection of proximal and distal arms between R_4+5_ and M (1 = present, 2 = absent). *Thorax*: M1, maximum length; M2, width at the level of the postsutural supra-alar seta; and M3, diagonal distance from the postsutural-supra-alar seta to the apex of the scutellum. In addition, ratios were used, X1, proportion of the total length of the aculeus and maximum length of the thorax (A1/M1); X2, proportion of the total aculeus length and wing length from the basal extreme of the costal vein to the apex (A1/W1); and X3, proportion of the maximum thorax length and maximum wing length (M1/W1).

### Statistical analysis

The samples were grouped according to their geographical origin to discern the degree of variability or possible differentiation between populations. The mean and standard deviation were calculated for each of the variables. A discriminant function analysis (DFA) was applied to the set of variables. Measures of the mean distances of the data were derived from the comparison of pairs of centroids, expressed as the Mahalanobis distance (MD). The functions were evaluated by applying a canonical correlation analysis to determine the significance and discrimination power of the model and the specific variables responsible for the segregation of the groups. All statistical analyses were conducted using the software Statistica 12 (StatSoft Inc. 2014). The *voucher* specimens of the Colombian, Peruvian and Brazilian populations were deposited in the Entomology Collection Laboratory of the University of Tolima, Ibagué –Tolima, and the samples from Mexico and Dominica were deposited in the Entomological Collection of the Institute of Ecology (INECOL), Xalapa – Mexico.

## Results

### Colombian populations

The results of the DFA applied to 11 Colombian populations of *Anastrepha obliqua* showed significant differences among them (Wilks’ Lambda: 0.00444, F (180,1498) = 7.0895, p < 0.0001). The exploratory model included 23 variables assessed, and 15 of them had a significant contribution to the model (P < 0.05): aculeus (A2, A3, A4, A7, A8, A9 and A10), wing (W1, W3, W4 and W5) and mesonotum (M1 and M2); significant differences in the ratios of X1 and X2 were also observed (Table 2).

**Table 2.** Discriminant function analysis summary of 11 Colombian populations of *Anastrepha obliqua*. Only significant variables in the model are included.

Variables	Wilks’ Lambda	F-remove 10,167	p-level	R-Square
W4	0.006767	8.825243	< 0.0001	0.280973
A2	0.005525	4.120675	< 0.0001	0.521245
A9	0.005423	3.736215	0.000151	0.990530
A4	0.005394	3.627670	0.000215	0.995470
W3	0.005309	3.305551	0.000614	0.101540
M2	0.005235	3.023529	0.001528	0.631673
M1	0.005153	2.713091	0.004108	0.987195
A8	0.005138	2.655682	0.004922	0.995699
W5	0.005116	2.572283	0.006392	0.118329
X1	0.005097	2.502542	0.007942	0.988528
A3	0.005069	2.394307	0.011094	0.644490
X2	0.005058	2.353962	0.012554	0.996985
W1	0.005055	2.343680	0.012954	0.996412
A7	0.005029	2.243795	0.017544	0.278160
A10	0.004946	1.927770	0.044553	0.986000

The Chi-squared tests indicated that the first eight, of the resulting ten canonical roots, were significant. Based on the standardized coefficients for the significant morphological variables, the first canonical root (CV-1) represented 63.1% of the model discrimination, CV-2 represented 13.5% and CV-3 represented 7.2% (Table 3).

**Table 3.** Standardized coefficients for canonical variables resulting from the discriminant function analysis of 11 Colombian population of *Anastrepha obliqua*. All canonical roots were significant.

Variables	Root 1	Root 2	Root 3	Root 4	Root 5	Root 6	Root 7	Root 8
A2	0.391	-0.131	-0.453	-0.417	-0.136	0.526	0.020	-0.392
A8	0.970	2.180	2.065	-5.627	-2.515	-5.268	-4.700	-1.553
M2	-0.396	0.555	0.263	0.021	-0.230	-0.093	0.094	-0.243
W4	0.021	-0.272	-0.887	0.073	0.103	-0.369	0.060	0.352
A10	-0.798	-1.386	-0.290	3.342	-1.684	0.533	0.084	-0.658
W3	0.084	0.006	-0.446	0.271	-0.064	0.049	-0.442	-0.009
A4	0.346	-0.572	-2.466	4.026	5.605	6.379	6.264	2.871
A9	0.480	0.467	-1.652	2.147	4.136	4.178	4.718	2.849
W2	0.165	-0.241	0.056	-0.037	0.317	0.647	0.377	-0.686
W5	0.038	0.072	0.022	0.088	0.334	0.333	-0.522	0.410
A3	0.382	-0.141	0.422	-0.092	-0.384	-0.286	0.126	0.669
A7	-0.252	0.222	-0.064	0.011	0.318	0.099	0.314	0.207
M3	0.274	0.574	-0.672	-0.455	0.127	0.629	-0.436	0.095
X2	-0.038	-5.365	1.610	3.953	-1.078	-2.621	-1.080	-9.026
W1	0.030	-4.933	1.722	3.171	-0.856	-2.958	-1.380	-8.032
M1	-1.661	2.220	-1.475	-1.368	-0.243	-0.342	3.662	0.908
X1	-1.273	2.550	-1.813	-1.511	-0.398	0.184	3.680	0.877
A1	0.657	1.293	0.231	-0.453	0.740	0.977	-0.878	3.841
Eigenvalue	7.660	1.637	0.891	0.618	0.488	0.350	0.233	0.141
Cummulative %	0.631	0.766	0.839	0.890	0.930	0.959	0.978	0.990

**Figure 3.**
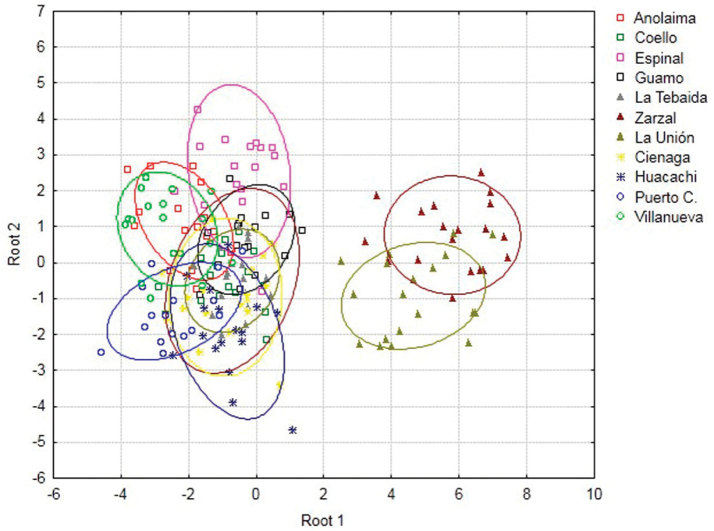
Discriminant function analysis applied to 11 Colombian populations of *Anastrepha obliqua*. Centroids with 95% prediction ellipses.

**Figure 4.**
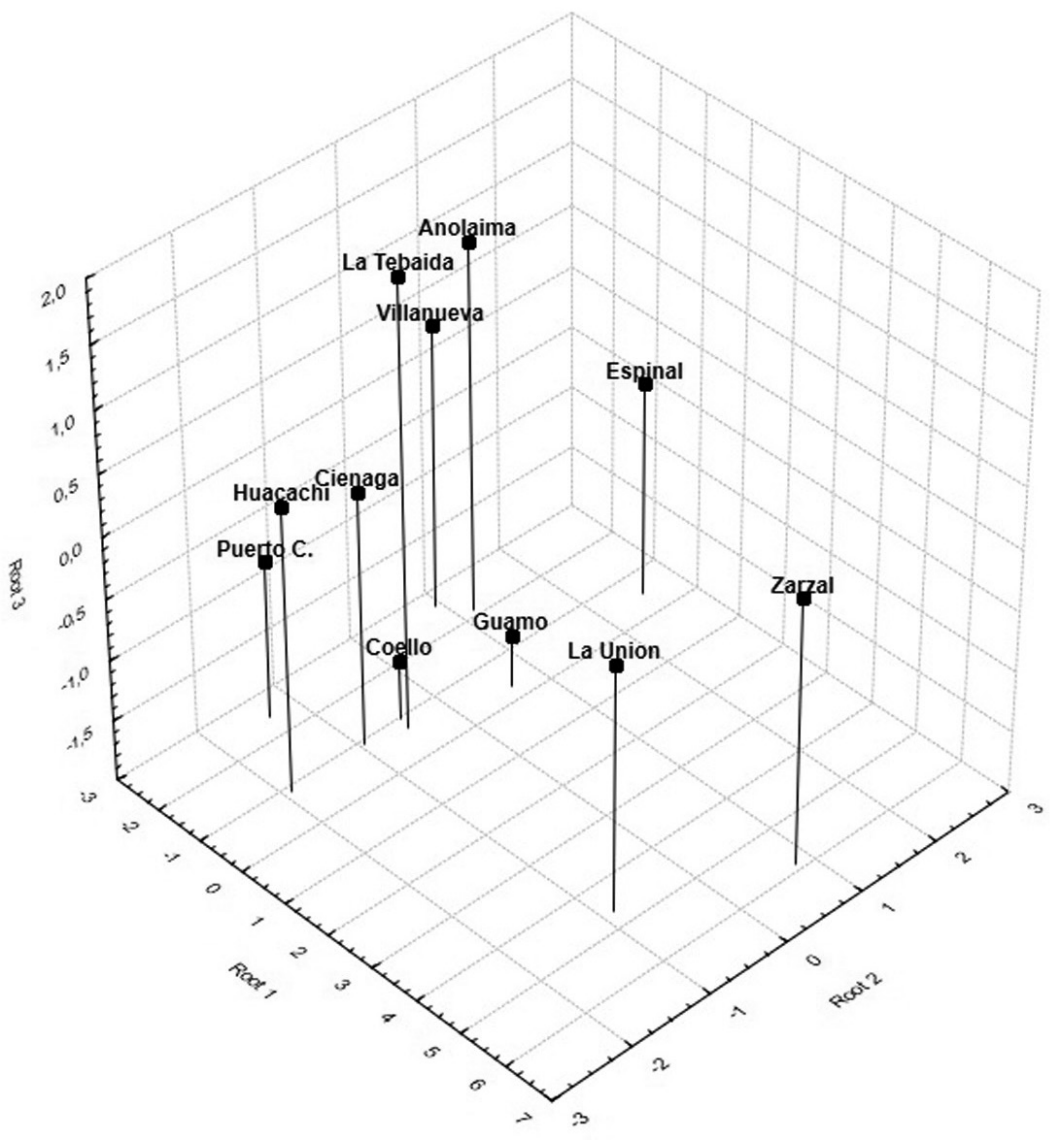
3D scatterplot of discriminant function analysis of 11 Colombian populations of *Anastrepha obliqua*.

The scatterplot obtained with the first two discriminant functions (Root 1 and Root 2) indicates the formation of two distinct groups. The first group was composed by nine populations distributed along the middle Valley and lower Magdalena River (Anolaima, Coello, Espinal, Guamo, Cienaga, and Huacachi), one sample in the Andean basin of the Cauca River (La Tebaida), and two in the eastern Plains (Villanueva and Puerto Colombia). The second group was composed by two populations located in the Cauca River Valley (La Unión and Zarzal) (Figure 3). The 3D scatterplot based on the first three canonical roots shows the mean distances between the centroids of each of the populations (Figure 4), where La Unión and Zarzal are located distally on canonical axis 1 (positive coefficient), while other populations are located basally to this axis (negative coefficient).

**Figure 5.**
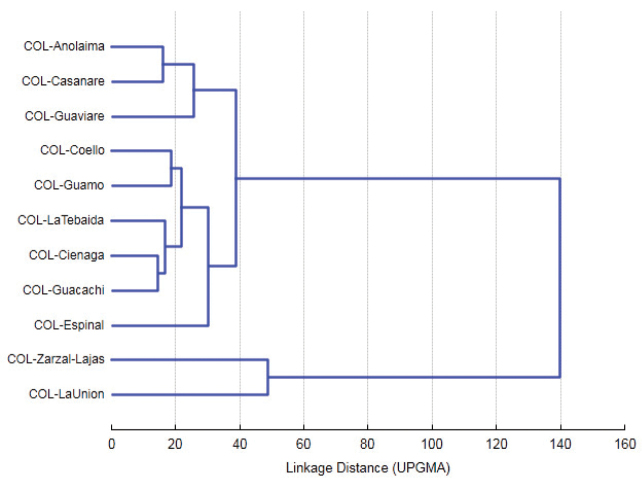
Dendrogram of morphological similarities based on the distance matrix between 11 Colombian populations of *Anastrepha obliqua*.

The largest Mahalanobis distances were found between Zarzal and La Unión with respect to the other populations (MD = 45.38–76.04 and MD = 32.89–58.3, respectively); the other nine populations had distances between 5.47 and 23.27 (Fig. 5).

The grouping of 11 Colombian populations based on the means of all of the variables resulted in the formation of two large clusters (Euclidian Distance=142): one included the Zarzal and La Union populations, and the second comprising the remaining nine populations (Figure 5).

### Colombian and external populations

The comparison of the Colombian populations with the external populations was conducted by sorting the Colombian populations into two groups according to our previous results. The first (Colombia-1) included the populations of the Magdalena Valley, the Coast and the eastern Plains, and the second (Colombia-2) included the individuals of La Unión and Zarzal. Discriminant analysis was performed for all 23 variables; however the W5 and W6 put too far the Brazilian populations and hindered the variability among other groups. Hence, a DFA was applied with 21 morphometric variables (excluding W5 and W6), and a significant differentiation was found among the groups (Wilks’ Lambda: 0.00596, F (119,1724) = 17.300; p < 0.0001). This model was constructed with the 17 variables resulted of significance (Table 4). The analyses of the standardized coefficients produced seven significant discriminant functions and the first three represented 82.9% of the discrimination among groups (Table 5).

**Table 4.** Discriminant function analysis summary of *Anastrepha obliqua* grouped by Colombian morphos and other Neotropical samples. Only significant variables in the model are included.

Variables	Wilks’ Lambda	F-remove (7,263)	p-level	R-Square
W3	0.009257	20.74111	< 0.0001	0.160514
W1	0.008741	17.49202	< 0.0001	0.997393
A1	0.008487	15.89532	< 0.0001	0.987234
X2	0.008162	13.84414	< 0.0001	0.996066
W4	0.007055	6.87049	< 0.0001	0.188851
A10	0.006982	6.41526	< 0.0001	0.959241
M1	0.006709	4.69552	0.000056	0.997781
X3	0.006672	4.46242	0.000104	0.996826
A7	0.006670	4.44466	0.000109	0.248941
A8	0.006569	3.80834	0.000586	0.993947
W2	0.006541	3.63394	0.000927	0.735419
A3	0.006487	3.29340	0.002249	0.637386
M3	0.006421	2.87547	0.006557	0.830076
M2	0.006406	2.78386	0.008263	0.630219
A2	0.006381	2.62708	0.012231	0.586167
A9	0.006371	2.56574	0.014242	0.987284
A5	0.006291	2.05764	0.048489	0.993312

**Table 5.** Standardized coefficients for canonical variables resulting from the discriminant function analysis of two Colombian population groups and other samples from the Neotropics of *Anastrepha obliqua*. All canonical roots were significant.

Variables	Root 1	Root 2	Root 3	Root 4	Root 5	Root 6	Root 7
W3	-0.14546	0.57827	-0.48720	-0.13891	-0.27142	-0.10810	-0.3763
W1	2.34202	8.07865	1.55013	10.35805	7.48457	-1.07338	6.4754
A1	0.23325	-5.10279	-1.59967	-2.67578	-1.76714	-0.32702	0.0577
X2	0.14063	8.22717	1.60284	6.11910	3.52443	0.54381	-1.0280
W4	0.05219	-0.04958	-0.45978	-0.15183	-0.04098	0.49208	0.0617
A10	-0.26785	-0.34056	-1.26399	1.73401	1.06799	0.34111	-2.5677
M1	-2.99532	-0.23069	0.69352	-5.98948	-6.33550	2.14080	-10.3391
X3	2.23303	0.27413	-0.47150	5.02686	4.85777	-2.58086	8.6129
A7	-0.21248	-0.17495	-0.31814	0.07865	-0.22900	-0.15917	0.1443
A8	-0.32696	-0.54133	3.10445	-3.08459	-3.72604	-1.88189	1.5067
W2	0.05675	-0.09495	-0.36750	-0.16253	-0.36755	-0.80740	0.5985
A3	0.39987	0.27838	-0.02253	-0.16642	0.18564	0.16871	-0.2246
M3	0.06434	-0.25025	-0.11419	-0.25789	0.99853	0.42983	-0.1847
M2	-0.21312	0.03360	-0.09815	0.52113	-0.19375	0.09046	0.1040
A2	0.34622	-0.05214	0.04938	-0.01043	-0.37470	0.23335	0.0871
A9	0.70756	0.89411	-1.52496	0.14320	2.86986	0.96121	1.2761
A5	1.00339	1.01537	-1.71105	1.29513	3.46539	1.19083	0.5293
Eigenvalue	5.18751	2.05775	1.23376	0.89666	0.40178	0.31538	0.1344
Cummulative %	50.7	70.8	82.9	91.7	95.6	98.7	100.0

**Figure 6.**
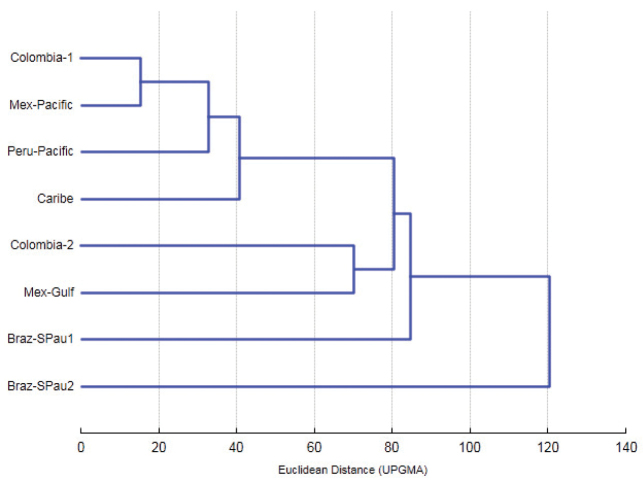
Dendrogram based on the Euclidean distance of Colombian groups of *Anastrepha obliqua* compared with six Neotropical populations using the UPGMA method.

The grouping of samples based on the means of all the variables and using the first two canonical roots showed the divergence between the Colombian groups. Colombia-1 was placed in a group with the Mex-Pacific, Peru-Pacific and Caribbean populations; Colombia-2 formed a group with the Mex-Gulf population, and the Brazilian samples remained separate (Figure 6).

**Figure 7.**
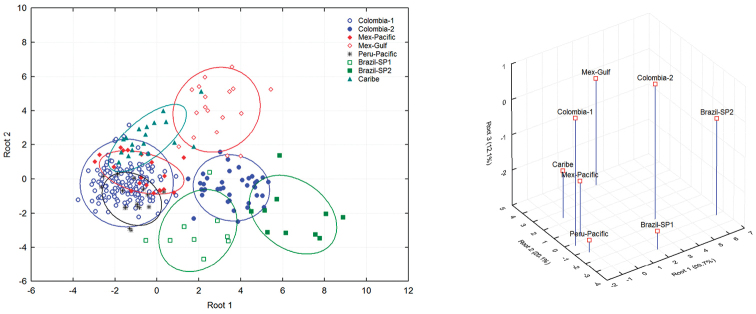
Discriminant function analysis applied to morphometric variables of females of Colombian *Anastrepha obliqua* populations compared with six external populations. **A** Scatterplot of individuals showing centroids with 95% prediction ellipses **B** Mean distance among centroids (3D scatterplot).

The scatterplot obtained by the comparison of the first two discriminant functions (Root 1 and Root 2) and the 3D scatterplot based on the first three canonical roots are shown in Figure 7.

The prediction model was able to correctly assign 93.4% of the individuals in their corresponding groups; all of the Brazilian individuals were classified correctly, and the success rates of the other groups were between 89.5 and 97.4% (Table 6). The model indicates that the Colombian samples were correctly separated into two groups, as the incorrectly assigned individuals in each of the Colombian groups were located in groups from other countries.

**Table 6.** Classification matrix of individuals according to a predictive model of two Colombian groups and six Neotropical samples of *Anastrepha obliqua*. Rows: Observed classifications; Columns: Predicted classifications. Same probabilities for all the groups.

	% Correct	Colombia-1	Colombia-2	Mex-Pacific	Mex-Gulf	Peru-Pacific	Brazil-1	Brazil-2	Caribe	N
Colombia-1	91.8	**145**	0	10	0	3	0	0	0	158
Colombia-2	97.4	0	**37**	0	1	0	0	0	0	38
Mex-Pacific	94.7	1	0	**18**	0	0	0	0	0	19
Mex-Gulf	94.4	0	0	1	**17**	0	0	0	0	18
Peru-Pacific	93.8	0	0	1	0	**15**	0	0	0	16
Brazil-1	100.0	0	0	0	0	0	**9**	0	0	9
Brazil-2	100.0	0	0	0	0	0	0	**10**	0	10
Caribe	89.5	1	0	0	0	1	0	0	**17**	19
Total	**93.4**	147	37	30	18	19	9	10	17	**287**

The means and standard deviations for each of the variables studied are shown in Table 7.

**Table 7.** Means and standard deviations (mm) of morphometric variables of two Colombian groups of *Anastrepha obliqua* females and six other populations from the Neotropics. Values of A9, A10, A11, X1, X2, X3 represent ratios of two variables, W5 and W6 refer to presence/absence.

Variables	Colombia-1	Brazil-1	Brazil-2	Mex-Pacific	Mex-Gulf	Peru-Pacific	Caribe	Colombia-2
A1	1.52±0.04	1.70±0.02	1.72±0.04	1.56±0.05	1.56±0.03	1.60±0.05	1.5±0.03	1.62±0.04
A2	0.08±0.00	0.09±0.01	0.10±0.01	0.08±0.01	0.10±0.01	0.08±0.00	0.09±0.01	0.10±0.01
A3	0.07±0.00	0.08±0.01	0.09±0.01	0.07±0.00	0.08±0.01	0.06±0.00	0.07±0.01	0.08±0.00
A4	0.05±0.01	0.04±0.01	0.06±0.00	0.05±0.01	0.05±0.01	0.06±0.01	0.06±0.01	0.06±0.01
A5	0.11±0.01	0.12±0.01	0.14±0.01	0.12±0.01	0.13±0.00	0.11±0.01	0.11±0.01	0.14±0.01
A6	0.17±0.01	0.17±0.01	0.21±0.01	0.17±0.01	0.19±0.01	0.18±0.01	0.17±0.01	0.20±0.01
A7	10.6±0.98	11.56±0.39	11.03±0.64	11.47±0.66	10.78±0.89	10.5±1.08	10.05±0.76	10.39±0.89
A8	0.16±0.01	0.16±0.01	0.20±0.01	0.17±0.01	0.19±0.01	0.17±0.01	0.17±0.01	0.19±0.01
A9	0.42±0.08	0.36±0.07	0.41±0.05	0.39±0.05	0.39±0.05	0.57±0.07	0.53±0.06	0.42±0.06
A10	0.12±0.01	0.12±0.01	0.14±0.01	0.12±0.01	0.14±0.01	0.13±0.01	0.13±0.01	0.14±0.01
A11	1.11±0.01	1.12±0.01	1.14±0.01	1.12±0.01	1.13±0.00	1.11±0.01	1.11±0.01	1.14±0.01
W1	6.62±0.31	6.9±0.41	7.27±0.15	6.86±0.16	7.44±0.19	6.68±0.27	6.76±0.33	6.84±0.27
W2	2.7±0.14	2.77±0.18	3.01±0.06	2.90±0.07	3.03±0.11	2.78±0.15	2.83±0.14	2.77±0.12
W3	0.45±0.05	0.43±0.1	0.45±0.05	0.57±0.04	0.60±0.07	0.52±0.05	0.61±0.05	0.47±0.06
W4	1.31±0.14	1.54±0.05	1.48±0.09	1.45±0.1	1.44±0.11	1.40±0.11	1.54±0.09	1.35±0.11
W5	1.03±0.18	1.11±0.33	1±0	1±0	1±0	1±0	1±0	1±0
W6	1.07±0.26	2±0	2±0	1.05±0.23	1±0	1±0	1±0	1.05±0.23
M1	3.07±0.2	2.95±0.2	3.05±0.11	3.24±0.11	3.18±0.16	3.25±0.13	3.00±0.17	2.96±0.18
M2	1.95±0.13	1.98±0.05	1.93±0.07	2.01±0.06	2.04±0.1	2.04±0.12	1.89±0.13	1.87±0.12
M3	1.93±0.13	1.89±0.06	1.95±0.09	2.01±0.08	2.02±0.1	2.09±0.11	1.92±0.13	1.88±0.13
X1	0.50±0.04	0.58±0.04	0.57±0.02	0.48±0.02	0.49±0.03	0.49±0.02	0.50±0.03	0.55±0.04
X2	0.23±0.01	0.25±0.02	0.24±0.01	0.23±0.01	0.21±0.01	0.24±0.01	0.22±0.01	0.24±0.01
X3	0.46±0.02	0.43±0.02	0.42±0.01	0.47±0.02	0.43±0.02	0.49±0.02	0.44±0.01	0.43±0.03
n	158	9	15	19	18	16	19	38

## Discussion

The morphometric analysis of natural populations of *Anastrepha obliqua* from Colombia resulted in the separation of individuals into two groups. The Zarzal and La Unión populations had the greatest values for ovipositor width at the end of the oviduct (A2, 0.09–0.11 mm); width at the beginning of the serrated section (A3, 0.08–0.09 mm); length of the tip of the aculeus (A4, 0.12–0.15 mm); length of the apex of the aculeus (A4+A5, 0.18–0.21 mm) and the proportion of the length of the tip of the aculeus and total aculeus length (A10, 0.13–0.15 mm). The remaining populations had smaller values: A2, 0.075–0.083 mm; A3, 0.06–0.08 mm; A4, 0.09–0.12 mm; A4+A5, 0.14–0.18 mm and A10, 0.11–0.13 mm. The tip of the aculeus is one of the most important taxonomic characters for species separation within the genus *Anastrepha* (Zucchi 2000, Hernández-Ortiz et al. 2004) and contributed here to separate the Colombian populations into two groups. The usefulness of the linear morphometry was shown for *Anastrepha fraterculus* collected from different countries in Latin America (Hernández-Ortiz et al. 2004, 2012, 2015) and, together with additional cytogenetic studies (Goday et al. 2006), reproductive isolation (Vera et al. 2006, Devescovi et al. 2014) and chemotaxonomy (Vaníčková et al. 2015) have demonstrated that it is a cryptic species complex.

The populations of the Sabana province (to the east) have no apparent morphological separation, although they form a clade with one Magdalena River population (Anolaima) that is slightly separated from the Magdalena river populations and the Magdalena (Cienaga) and Guajira (Huacachi) provinces (Euclidean distance≈ 40). These populations are also geographically isolated and should be studied more extensively.

Populations from the Cauca River Valley presented higher levels of variability. Two of them segregated into one group and La Tebaida population grouped with the other populations from the Magdalena River and the Caribbean and eastern region. This population is at the highest altitude surveyed and is isolated by the mountains from the smaller valleys that run into the Cauca River. Two of the authors (N. Canal and M.R. Castañeda) traveling along the Cauca River Valley found roadside mango markets without any type of sanitary control and whose product came from Tolima crops in the Magdalena Valley. One possible explanation for the similarity found, would be that these markets move infested fruit from the Magdalena valley to the Cauca river valley and some populations established in specific areas such as for La Tebaida population. In contrast the Zarzal and La Union populations were already collected in the Valley plains and may represent the local variability.

By including the six external populations in the discriminant analysis, the predictive model indicated that two Colombian groups remain isolated, reinforcing the result that there may be two groups in Colombia.

Genetic studies conducted by Ruíz-Arce et al. (2012) using COI and ND6 markers in Latin American and Caribbean populations of *Anastrepha obliqua* indicated that the Colombian and Peruvian populations formed a single genotype, which the authors called the Andean South American type; however, those authors included four Colombian populations, all belonging to the geographical Magdalena River Valley. The morphometric analysis also indicates that all the populations of the Magdalena River Valley are similar; divergent populations were those collected in the Cauca River Valley.

Predictive model showed in addition that the Mex-Pacific and Peru-Pacific populations are close to the Magdalena River populations (Colombia-1), forming a relatively compact group. Also, the Gulf of Mexico population (Mex-Gulf) is grouped with the Colombian populations of the Cauca River (Colombia-2), however, there is one important divergence represented by the high Euclidean distance between them (≈80), suggesting that it may correspond to a different group in accordance with the findings of Smith-Caldas et al. (2001) and Ruíz-Arce et al. (2012). The Caribbean (Dominica Island) population is grouped with the clade of Colombia-1 but was also slightly divergent. The Brazilian populations represent a group that is morphometrically different from all the other populations studied (Euclidean distance ≈120); however, the distance between them is high (≈85).

Genetic studies conducted previously indicate the existence of those groups and established some relationship among them. Smith-Caldas et al. (2001) found that the populations of *Anastrepha obliqua* studied with COI were combined into two groups; the population of Colombia was included in a group with a population of Brazil (northeast) and a sample of Los Tuxtlas (Gulf of Mexico); the second group included populations of Brazil. This finding was similar to the results of the linear morphometrics. Ruíz-Arce et al. (2012) found that eastern populations of the Sierra Madre in Mexico were genetically similar to the populations of Colombia (four from the Magdalena River) and Peru and that the populations of the eastern Sierra Madre (Tuxtlas) were separate from them. The morphometric results confirm these findings. Likewise, Ruiz-Arce et al. (2012) found that the samples that came from the Caribbean Islands had variability with respect to the other samples.

Ruíz-Arce et al. (2012) included nine populations of southeastern and northeastern Brazil, and all the haplotypes found corresponded to the same group. In contrast, Smith-Caldas et al. (2001) found that the genotypes of populations of southeastern Brazil formed a very strong, separate group from the other group that included a northeastern population. Our studies included only two populations of southeastern Brazil that had a morphometric divergence. Morphometric studies of *Anastrepha obliqua* should be expanded to include a greater number of populations across a wider range of the species distribution.

The morphometric analyses of *Anastrepha obliqua* females indicate that in Colombia there could be two different morphotypes and also that the external samples could be divergent and several groups may exist. Larger studies should be performed to confirm this hypothesis.

Traditionally, linear measurements have been valid tools to separate species within the existing diversity; however, the development of modern tools in morphometry, genetics, behavior and ecology has allowed the recognition of a wide variability within the existing nominal species, suggesting the existence of greater diversity in nature and hampering the definition and delimitation of the species (Baylac et al. 2003, Bickford et al. 2007, Wiens 2007, de Queiroz 2007, Yeates et al. 2011, Krosch et al. 2013). De Queiroz (2007) recognizes that there are many existing definitions of species and that many of them exist due to the tools and particular interests of the researchers. In some cases, as when speciation is more incipient, the existing tools are more inefficient. Thus, in the modern delimitation of species, the iterative or integrative proposal of taxonomy appears as the best approach (Yeates et al. 2011).

Following the proposal by De Queiroz (2007) and Yeates et al. (2011), the studies by Smith-Caldas et al. (2001), Ruíz-Arce et al. (2012) and our group with molecular markers and morphometry suggest the presence of seven groups within the species, whose denomination may be a modification of that proposed by Ruíz-Arce et al. (2012), Mesoamerica, Central America, Caribbean, western Mexico, Magdalena, Cauca and Brazil, which must be at the very least, separate metapopulations lineages. Other biological, ecological and morphological studies are needed to define the definitive limit of these lineages as species.

## Conclusions

The results of this work, in conjunction with previous studies, indicate that in Colombia, two groups exist that could be under a divergence process which could lead to speciation. Further, the same seems to be occurring in different Neotropical regions. Larger studies are required to define the taxonomic status of the species, which could be relevant for pest management.
